# Analysis of Eating Habits and Body Composition of Young Adult Poles

**DOI:** 10.3390/nu13114083

**Published:** 2021-11-15

**Authors:** Anna K. Mazurek-Kusiak, Agata Kobyłka, Natalia Korcz, Małgorzata Sosnowska

**Affiliations:** 1 Department of Tourism and Recreation, University of Life Sciences in Lublin, Akademicka 13, 20-950 Lublin, Poland; anna.mazurek@up.lublin.pl; 2 Department of Natural Foundations of Forestry, Institute of Soil Science and Environment Management, University of Life Sciences in Lublin, Akademicka 13, 20-950 Lublin, Poland; natalia.korcz@up.lublin.pl; 3 Department of Grassland and Landscape Shaping, University of Life Sciences in Lublin, Akademicka 13, 20-950 Lublin, Poland; malgorzata.sosnowska@up.lublin.pl

**Keywords:** eating habits, young adults, Poland, obesity, overweight, barriers, motivations, education, body composition analyzer

## Abstract

Background: Obesity and overweight affect a large proportion of the world’s population. Increasingly, this problem can be observed among young adults. The aim of the study was to identify the motivations and barriers to healthy eating habits among young Poles, the relationship between physical activity and healthy eating and the impact of healthy eating on the body composition of the young. Methods: The method used in the research was a diagnostic survey using direct personal interviews. The research was conducted in the years 2016–2019 on a group of 399 young Poles aged 18–26. Their body composition was analyzed by determining resistance and reactance using the biological impedance method, with a TANITA SC-330ST Body Composition Analyzer. Results and conclusion: The main reasons for healthy eating among young Poles are the intent to follow a doctor’s recommendations, to lose weight and to live a healthy lifestyle and to follow a trend. On the other hand, the largest barriers to proper nutrition are: lack of time to prepare healthy meals and of financial resources, inability to prepare meals and limited knowledge of the principles of healthy eating. The eating behavior varied significantly in relation to the physical activity of the respondents. Active people’s eating habits were the best, and those of sedentary people the worst. Healthy eaters also had normal body composition indicators (adipose tissue level, BMI, body type). Young adults should be educated on the principles of healthy eating and have access to healthy food in canteens and vending machines, both at work and at university.

## 1. Introduction

A global study on nutrition conducted in 195 countries by more than 130 scientists from 40 countries showed that statistically every fifth person in the world dies from unhealthy eating [1]. As reported by Wang et al., an important goal of the Asian National Health Promotion Movement in the 21st century is to increase the consumption of vegetables and fruits and maintain a balanced diet. In addition, the organization reports the need to monitor eating patterns in societies [2]. Consumption of food according to nutritional standards aims to prevent diseases associated with both energy and nutrient deficiencies and the harmful effects of excess [3]. Eating organic, non-chemically processed foods is also important [4]. 

In Poland, in 2016, the National Food and Nutrition Institute published a new pyramid of Healthy Nutrition and Physical Activity based on the latest scientific research and recommendations of global expert centers [5]. It differs significantly from the 2009 nutritional pyramid, because its base is constituted by physical activity understood not only as a typical sport, but also as taking the stairs, walking, Nordic walking, etc. Physical activity as the base of the pyramid was already included in the 2009 version, but now it has been assigned much greater importance. According to the National Institute of Food and Nutrition, one should spend at least 30–45 min on physical activity every day [5].

A proper diet is closely related to physical activity, and both of them are of great importance in health prophylaxis. Properly selected forms of workout and the combination of the quantity and quality of meals prevent various illnesses [6,7,8,9,10,11,12,13,14]. 

Proper nutrition is one of the most important environmental factors influencing human growth, development, and the maintenance of good health. As Mazurek-Kusiak et al. [15] and Farrow et al. [16] point out, eating behaviors are formed in childhood and consolidated in adolescence. Young adults leaving the family home often have no correct eating habits. Moreover, a fast pace of life, professional work, studies, time constraints and lack of adequate financial resources, as well as stress and distress are the reasons why the “fast food” menu dominates the diet of young adults [17,18,19]. Furthermore, eating out means that the servings contain less fiber [20] and more calories, saturated fats, and cholesterol, which negatively affects the body composition of consumers [21]. Another problem is skipping breakfast [22,23] or too low consumption of vegetables and fruit [24]. Mledrum et al. [25] indicates that the lack of diversity in nutrition, insufficient number of meals and their irregularity, as well as snacking between meals is also a significant problem.

The rates of overweight and obesity have increased dramatically in recent decades [26,27], and the prevalence of overweight and obesity is higher in young adults than in other age groups [27,28]. Lobstein and Brinsden [29] report that 1307 million adults worldwide are overweight and 671 million obese. This problem also affects European countries [30], including Poland [31,32], where almost half of Poles (59%) have a problem with excess weight, including one fifth with obesity (21%) [33]. Therefore, investigating the reasons for employing healthy eating habits among young adults is justified and, even more so, pointing out the barriers that prevent Poles from following a healthy eating style. A discussion on this issue in national contexts is also recommended. Understanding the motivations and barriers to healthy eating habits will allow the development of effective policies and management programs in combating health problems (from obesity to anorexia). One of the strategic goals of the National Health Program for 2021–2025 in Poland is prevention of overweight and obesity [34]. This goal is strongly justified, as Polish society is characterized by poor eating habits and lack of physical activity. A better understanding of the relationship between diet and health is important for the development of behavioral change programs and strategies to improve lifestyle in general and, in particular, to reduce diet-related diseases [35].

The study hypothesized that people with proper eating habits have a positive body composition, with an adequate amount of fat and water in the body. The aim of the study was to identify the motivations and barriers to healthy eating habits among young Poles, the relationship between physical activity and healthy eating, and the impact of healthy eating on the body composition of young adults.

## 2. Materials and Methods

### 2.1. Sample and Study Design

The method used in the research was a diagnostic survey using direct personal interviews (PAPI—paper and pencil interview). The interview questionnaire consisted of 3 parts. The first part included personal data questions, to specify the characteristics of the respondents (gender, age, place of residence). The second part included questions on eating behaviors according to the recommendation of Starzyńska’s test [36], as well as motivations and barriers to healthy eating and physical activity. The third part included data on body composition and was filled in based on the results obtained from the analyzer. The research was conducted in the years 2016–2019 on a group of 399 young Poles aged 18–26, in different areas of Poland. The sampling was random, taking into account the sampling of Polish society in terms of gender (women—225, men—174) and type of place of residence (villages—140, towns with up to 20,000 residents—119, cities with over 20,000 residents—140). The established number of respondents was representative for individual regions of the country: north-east—100; north-west—100, south-east—100 and south-west—99 (Table 1). 

The assessment of eating habits was carried out using the modified Starzyńska’s test (Table 2 and Table 3) concerning, e.g., the number of meals during the day and the frequency of consumption of individual nutrients [36].

The research was supplemented by a survey on the motivations and barriers to following healthy eating habits and the strength of the impact of individual factors on a healthy eating style, using a 5-point Likert scale, where 1 meant “it does not matter”, 2 meant “it has little importance, 3 meant “it is of medium importance”, 4 meant “it is important” and 5 meant “it is very important”. The questionnaire on motivations and barriers to healthy eating habits was validated before completion.

The level of physical activity was also assessed using a direct interview questionnaire. Respondents were asked to self-assess their physical activity on a nominal 4-point scale, where 1 meant inactive (sedentary lifestyle, lack of physical activity), 2—not very active (physical activity related to walking), 3—active (moderate activity) and 4—very active (intense activity). Intensive physical activity is understood as strenuous physical exertion causing rapid breathing, increased heart rate and sweating of the body. The term “moderate physical activity” describes average physical exertion with slightly increased breathing, slightly increased heart rate and not necessarily sweating. 

Body composition analysis was carried out by determining the resistance and reactance values based on the biological impedance method, using a TANITA SC-330ST Body Composition Analyzer.

The device has a built-in scale and a thermal printer. Analysis is a reliable, non-invasive, safe and effective method of testing body composition compared to classical anthropometric techniques [38,39,40]. During the test, the person stood with their bare feet on the measuring platform, with their feet touching the metal electrodes. The measurements were carried out under the recommended conditions (following a morning fast). Respondents also declared not having consumed strong tea, caffeine, alcohol or medications for at least 24 h prior to the study.

The following measurements were made using the analyzer: weight [kg], BMI [kg/m^2^], FAT [%], TBV [%], physique rating (the ratio of body fat and muscle mass in your body) [41,42,43]. 

### 2.2. Statistical Analyses

Statistica 13.3 PL (StatSoft Inc., Krakow, Poland) was used for statistical calculations. The calculations were performed at the confidence level of 0.95, and the maximum error rate was set at 0.05. 

The motivations and barriers of healthy eating were determined using a logistic regression model expressed as a mathematical formula (Formula (1)). It describes the effect of qualitative variables *x*_1_, *x*_2_, …, *x*_k_ on the dichotomous variable *Y* defined by two values: 1—success or 0—failure [44].

(1) $$P(Y=1|x_{1},\;x_{2},\ldots ,\;x_{k})=\frac{e^{\left(\alpha_{0}+\sum_{i=1}^{k}a_{i}x_{i}\right)}}{1+e^{\left(\alpha_{0}+\sum_{i=1}^{k}a_{i}x_{i}\right)}}$$

where:

$\alpha_{i},\;i=0,\ldots ,k$—logistic regression coefficients,

$x_{1}$, $x_{2}$,…, $x_{k}$—independent variables.

The value of the coefficients was determined using the maximum likelihood method, and the significance of individual variables was tested using Wald’s statistic. Maximum likelihood parameter estimators have an asymptotic normal distribution and are asymptotically the most effective. Thus, in testing the statistical significance of parameters, the asymptotic Student’s *t*-test can be used for sufficiently large samples. The quality of the constructed models was assessed using the percentage of correctly classified cases.

The differences between the motivations and barriers of healthy eating depending on the physical activity of young Poles were examined using discriminant analysis. Discriminant analysis examines the differences between groups based on a set of selected independent variables, using the formula [45]:

(2) $${\left(\vec{x}-{\vec{\mu }}_{0}\right)}^{T}\overset{-1}{\underset{0}{\sum }}\left(\vec{x}-{\vec{\mu }}_{0}\right)+\ln|\underset{0}{\sum }|-{\left(\vec{x}-{\vec{\mu }}_{01}\right)}^{T}\overset{-1}{\underset{1}{\sum }}\left(\vec{x}-{\vec{\mu }}_{1}\right)-\ln|\underset{1}{\sum }|>T$$

where: 

$w_{i\;}$—regression coefficients, 

${\vec{\mu }}_{k}$—mean parameters, 

$\sum_{k\;}$—covariance,

T—constant.

To examine the relationship between nominal and ordinal variables, crosstabs were used to synthetically present the emerging relationships using the χ^2^ measure, Pearson’s contingency coefficient C and gamma statistics.

Structural equation modeling was also used, as it estimates the multiple and interrelated dependences in a single analysis.

Correspondence analysis (CA), which is one of the multidimensional exploratory techniques, was also used to analyze the results. It allows summarizing the set of qualitative data (nominal and ordinal) in a two-dimensional graphic form [46]. The starting point for the analysis of such data is compiling them into a multi-way table. The observed numbers of simultaneous occurrences of R and C are recorded in a contingency table (Formula (4)):

(3) $$N=\left[n_{ij}\right]$$

where: 

*i* = (1, 2, …, *r*), *j* = (1, 2, …, *c*), 1 ≤ *i* ≤ *r,* 1 ≤ *j* ≤ *c*

*n_ij_*—number of units having the *i*-th category of the first variable (rows—R) and the *j*-th category of the second variable (columns—C).

The analysis of statistics and charts allows for inference regarding the relationships between the categories of variables, i.e., the columns and rows of the multi-way table. Each row and column of the correspondence table can be displayed in a c-dimensional space (properly r-dimensional) with coordinates equal to the values of the corresponding profiles. The row and column coordinates on each axis are scaled so that they have an inertia equal to the principal inertia along that axis; these are the principal row and column coordinates [46,47,48].

## 3. Results

### 3.1. Motivations and Barriers for the Use to Healthy Eating Habits among Young Poles

Proper nutrition is one of the most important environmental factors influencing human growth, development and the maintenance of good health. It is important throughout a lifetime, but the attitudes that develop at a young age deserve special attention.

In the first stage of the research, the respondents were questioned regarding the motivations and barriers of healthy eating habits. For this purpose, a logistic regression model with the binomial distribution and logit function as a link function was developed. The results of the logistic regression are presented in Table 4.

Evaluation—the coefficient of the multiple regression model; *t* (392)-value of the *t* statistic assessment of the significance of the estimated coefficients; *p*-value of the probability level for the *t*-test; χ^2^ Wald—Wald chi-square statistic value used to assess the significance of estimated parameters, *p* (for Wald)—*p*-level value Wald chi-square test; UDR—unit odds ratio. Source: own study based on research.

The *p* value for the χ^2^ statistic is highly significant (*p* < 0.001), so it can be concluded that doctor’s recommendation, the intent to lose weight and to live a healthy lifestyle, as well as following a trend have a significant impact on following healthy eating habits in one’s diet. Thus, the model can be described using the mathematical formula:

(4) $$P\left(Y=4\right)=\frac{e^{\left(-4.159+0.714x_{1}+0.6474_{x_{2}}+0.339x_{3}+0.452x_{4}\right)}}{1+e^{\left(-4.159+0.714x_{1}+0.6474_{x_{2}}+0.339x_{3}+0.452x_{4}\right)}}$$

Positive coefficients corresponding to the variables indicate that the increase of these values intensifies motivation to follow healthy eating habits. The one-unit odds ratio shows that a doctor’s recommendations regarding a healthy lifestyle has the greatest influence on logit function. If this determinant increases by 1 point, the probability of employing healthy eating habits will increase by as much as 104.2%. The other values can be interpreted in a similar way. Increasing the intent to lose weight by 1 point will result in the probability of applying healthy eating habits by 90.9%. Intention to lead a healthy lifestyle increases the chance of healthy eating by 40.4% and following a trend by 57.1% (Table 4). 

The percentage of correctly classified cases was used to assess the quality of the constructed model (Table 5).

Based on the results presented in Table 4, it can be concluded that the model correctly classifies 98.83% of the respondents who have healthy eating habits and 35.09% of those who do not (Table 5).

Evaluation—the coefficient of the multiple regression model; *t* (393)-value of the *t* statistic assessment of the significance of the estimated coefficients; *p*-value of the probability level for the *t*-test; χ^2^ Wald—Wald chi-square statistic value used to assess the significance of estimated parameters, *p* (for Wald)—*p*-level value Wald chi-square test; UDR—unit odds ratio. Source: own study based on research.

The *p*-value for the chi-square statistic is highly significant (*p* < 0.001). Therefore, it can be concluded that the variables *x*_1_, *x*_2_, *x*_3_, *x*_4_ constitute a significant barrier to healthy eating habits (Table 6). The mathematical model is expressed as:

(5) $$P\left(Y=3\right)=\frac{e^{\left(-0.369x_{1}-0.544x_{2}-0.156x_{3}-0.3862x_{4}\right)}}{1+e^{\left(0.369x_{1}-0.544x_{2}-0.156x_{3}-0.3862x_{4}\right)}}$$

The greatest barrier was the lack of financial resources (−0.544), which discouraged the respondents from purchasing wholesome products. The second barrier was the lack of time to prepare healthy meals (−0.369). The third barrier was the lack of knowledge about the principles of healthy eating (−0.386). A significant barrier was also the inability to prepare meals (−0.156) (Table 6).

The model correctly classifies 100.00% of respondents who follow healthy eating habits in their diets. Please note that the model correctly groups only 7.02% of respondents who are not healthy eaters (Table 7).

The model that achieved the most significant discriminating power was doctor’s recommendation (F = 14.522). The discriminant function also achieved high values for lifestyle (F = 11.634). An essential motivation for implementing healthy eating habits (F = 10.943) is the possibility of losing weight. The “self-care” category also obtained a relevant discriminatory power (F = 8.961). At *p* < 0.001, these motivations are the most significantly critical for physically very active persons. Definitely, the lowest value (F = 6.336) was observed in the case of the trend motivation. Importantly, this motivation is significantly more critical for physically active (0.612) and not active people (0.535) than other groups (Table 8).

When analyzing the barriers that make the application of healthy eating the most difficult, it was indicated that the highest value of F = 10.165 was achieved by the factor “no time”. This barrier is significantly more critical for not very active people (1.724) than for other groups. The discriminant function also achieved high values for the barrier associated with a lack of knowledge of the principles of healthy eating (F = 8.802). In this case, the classification function achieved almost three times higher values for very active persons than other people. However, the lack of financial resources (F = 6.492) is a significantly more critical barrier for physically inactive people (1.581) than for other groups. The discriminatory function was significantly the lowest for the barrier due to inability to prepare meals (F = 3.620). Physically active (1.685) and not active people (1.627) pay attention to this barrier the most often (Table 9).

**Figure 1:**
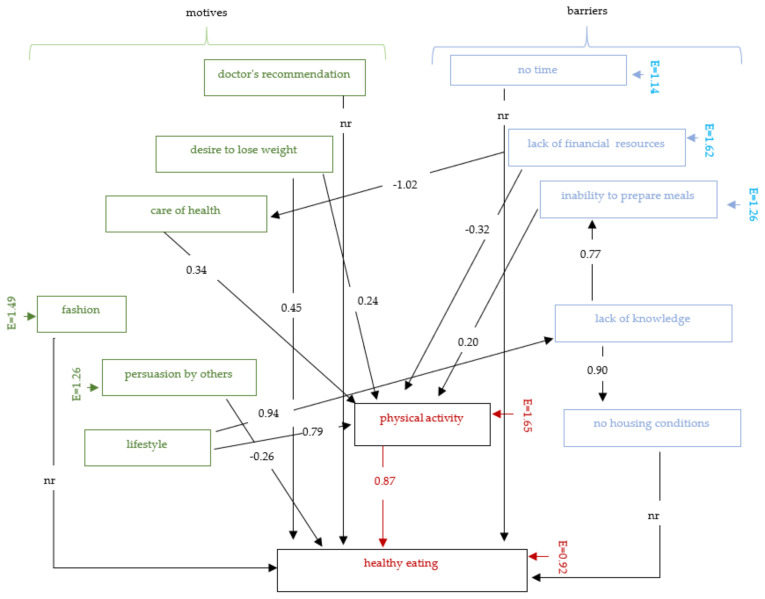
Graphical structural equation modelling: motivations and barriers to healthy eating. *nr—not relevant; E—residual variables*.

The structural equation model described indicates the relationship between motivations and barriers and physical activity and healthy eating. The four exogenous variables have no relationship with the endogenous variable: doctor’s recommendation, trend, lack of time, lack of appropriate housing conditions (Figure 1). 

### 3.2. Physical Activity and the Use of Healthy Eating Habits among Young Poles

In the next stage of the research, the eating habits of young Poles were confronted with their physical activity.

**Figure 2:**
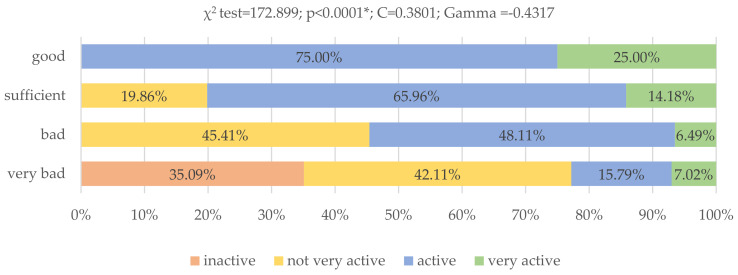
Assessment of nutritional behavior of young Poles and their physical activity (*N* = 399). * Statistically significant differences (*p* < 0.05). Source: own study based on the research.

The eating behavior varied significantly due to the physical activity of the young Poles (*p* < 0.0001, χ^2^ = 172.899). The C-Pearson contingency coefficient was 0.3801, which proves the average degree of correlation. Very active respondents (25.00%) and active (75.00%) had the best nutrition. As many as 35.09% young Poles who did not engage in any activity had very poor nutrition (Figure 2).

**Figure 3:**
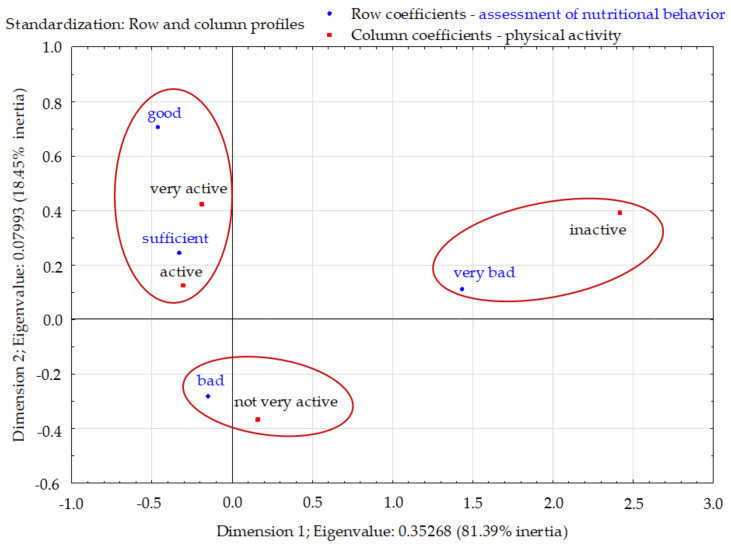
Assessment of nutritional behavior of young Poles and their physical activity (correspondence analysis). Source: own study based on the research.

The total inertia was 0.433 and indicated a fairly large scattering of the profiles. The profiles were mapped mainly in the first dimension (81.39%). Three clusters were clearly visible in the factorial space. The group of respondents with poor eating habits were dominated by inactive people. However, among the respondents with sufficient eating habits, active and very active people dominated. Respondents who had very poor eating habits differed significantly from the other three groups. On the other hand, respondents who were not physically active were not closely related to any group of eating behaviors, but they were closest to people with very poor eating habits (Figure 3).

### 3.3. Body Composition and the Use of Healthy Eating Habits among Young Poles

The nutritional condition of young adults was also analyzed in the context of the percentage of body fat, BMI and body type.

**Figure 4:**
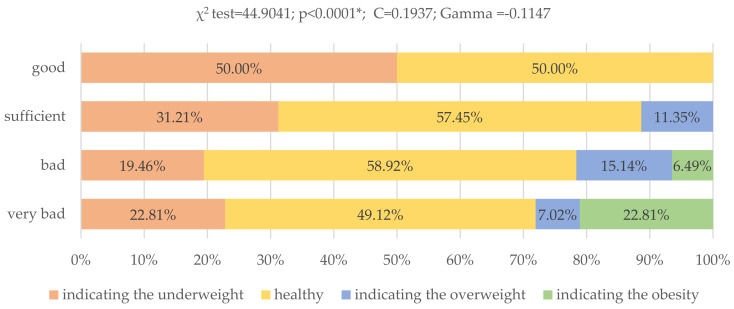
Assessment of nutritional behavior of young Poles and their level of body fat (*N* = 399). When assessing the level of body fat, the age and sex of the respondents were taken into account. * Statistically significant differences (*p* < 0.05). Source: own study based on the research.

Significant differences were also observed between body fat level and eating behaviors (*p* < 0.0001; χ^2^ = 44.904). The level of body fat indicating obesity was observed in up to 22.81% of respondents with very poor eating habits and in 6.49% of those with poor eating habits. On the other hand, the level of body fat indicating overweight was reported in 7.02% of the respondents with very poor eating habits, in 15.14% with poor habits and in 11.35% with sufficient eating habits. The level of body fat indicating either overweight or obesity was not observed in respondents following the dietary recommendations of Starzyńska (Figure 4).

**Figure 5:**
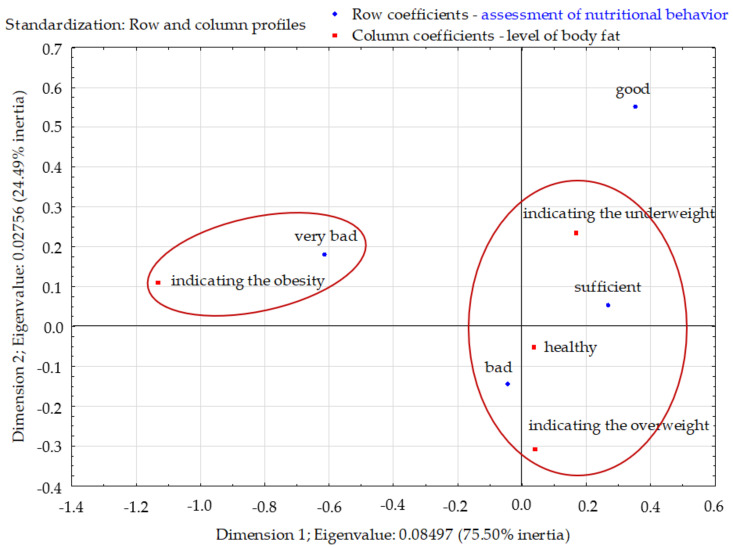
Assessment of the nutritional behavior of young Poles and the level of body fat in the organism (correspondence analysis). Source: own study based on the research.

The total inertia of the profiles was 0.123 and indicated a fairly large scattering. The profiles were mapped mainly in the first dimension (75.50%). Two clusters were clearly visible in the factorial space. Among respondents with very poor nutritional habits, the dominating group was those with the obesity level of adipose tissue. Respondents with sufficient and poor eating habits were characterized by the correct and excess level of body fat compared to other groups. Those with good eating habits did not differ from the other groups in terms of body fat level (Figure 5).

**Figure 6:**
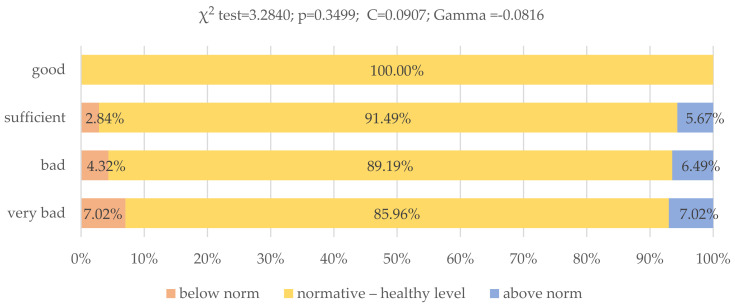
Assessment of nutritional behavior of young Poles and their level of total body water (*N* = 399). * When assessing the level of body fat, the gender of the respondents was taken into account. Source: own study based on the research.

Most of the respondents had the correct level of total body water. This percentage decreased along with the deteriorating eating habits of the respondents, but these differences were not statistically significant (*p* = 0.3499) (Figure 6). 

**Figure 7:**
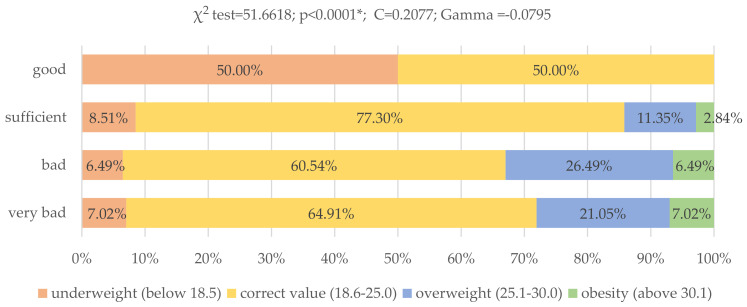
Assessment of nutritional behavior of young adult Poles and their level of BMI (*N* = 399). * Statistically significant differences (*p* < 0.05). Source: own study based on the research.

There were also significant differences between the studied groups of young adults and the BMI index (*p* < 0.0001; χ^2^ = 51.6618). The BMI of as many as 21.05% of respondents who did not comply with modified Starzyńska’s test recommendations indicated overweight and in 7.02% of them obesity. Among respondents with poor eating habits, as many as 26.49% were overweight, and 6.49% were obese. On the other hand, among respondents with a sufficient nutritional result, 11.35% were overweight and 2.84% were obese. The BMI of respondents following the dietary recommendations of the modified Starzyńska’s test indicated neither overweight nor obesity (Figure 7).

**Figure 8:**
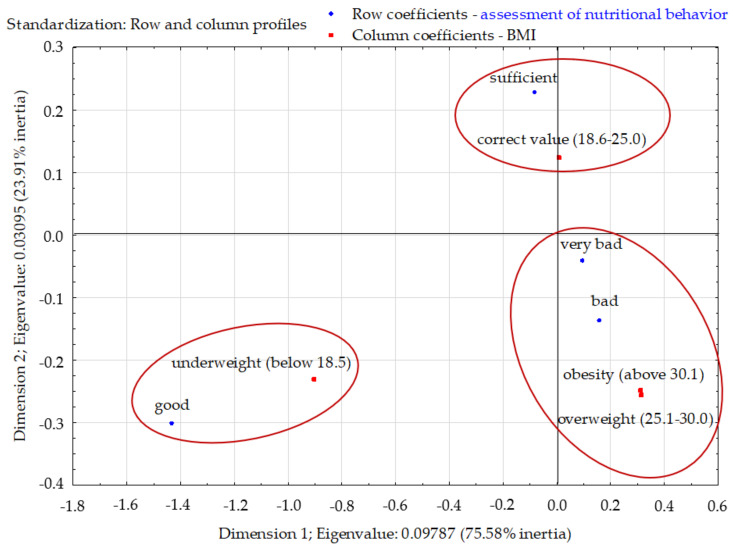
Assessment of nutritional behavior of young Poles and their level of BMI (correspondence analysis). Source: own study based on the research.

The total inertia of the profiles was 0.129 and indicated a fairly large scattering. The profiles were mapped mainly in the first dimension (75.58%). Three clusters were clearly visible in the factorial space. Among respondents with good eating habits, those with normal BMI prevailed. Respondents with poor and very poor eating habits stood out and distinguished themselves from the other two groups in terms of the percentage of BMIs indicating overweight and obesity. Respondents with good eating habits differed significantly from the other three groups (Figure 8).

**Figure 9:**
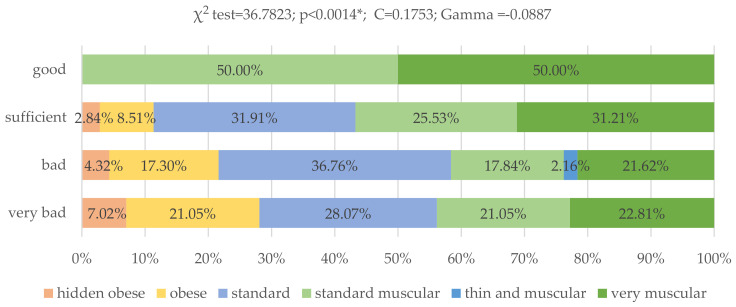
Assessment evaluation of the nutritional behavior of young Poles and their physique physical rating (*N* = 399). Age and sex of the respondents were taken into account when assessing body type. The study group did not include people with the following body types: solidly-built, under exercised, thin. * Statistically significant differences (*p* < 0.05). Source: own study based on the research.

There were also significant differences between the physique rating of the studied groups of young adults (*p* < 0.0014; χ^2^ = 50,2615). Obese body type was observed in 21.05% of respondents who did not comply with the modified Starzyńska’s test recommendations, in 17.30% of respondents with poor eating habits and in 8.59% with sufficient eating habits. Respondents who fully followed the modified Starzyńska’s test dietary recommendations had a very muscular (50.00%) and standard muscular (50.00%) body type (Figure 9).

**Figure 10:**
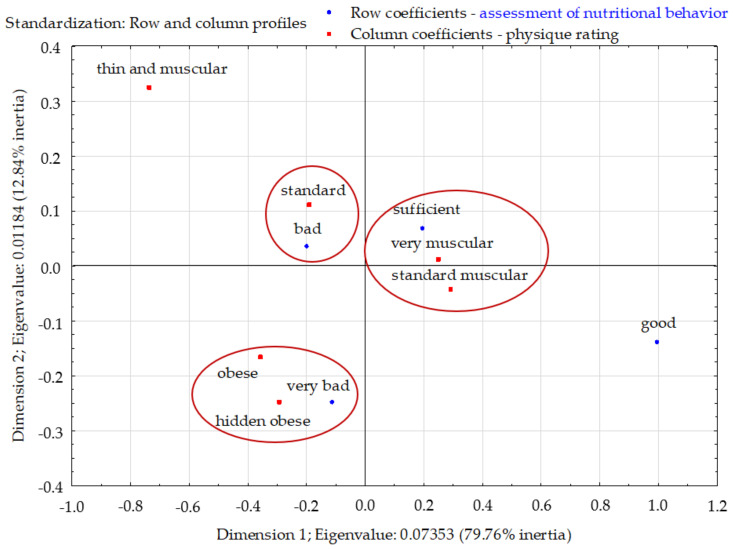
Evaluation of the nutritional behavior of young Poles and their physique rating (correspondence analysis). Source: own study based on research.

The total inertia of the profiles was 0.092 and indicated a low scattering. The profiles were mapped mainly in the first dimension (79.86%). Two clusters were clearly visible in the factorial space. Among respondents with very poor eating habits, obesity and hidden obesity dominated. Respondents with sufficient eating habits stood out, distinguishing themselves from other groups in terms of the percentage of standard muscular and very muscular people. Respondents with poor eating habits did not distinguish themselves from other groups in terms of the percentage of respondents with standard body build. Those with good eating habits were not closely associated with any body type (Figure 10).

## 4. Discussion

Eating habits are formed and developed during childhood and adolescence and then are transferred to adulthood. These habits are shaped by patterns and factors in the family home, peer group, at school, in the local community, media, etc. [49,50]. Research on eating habits is often conducted, but usually among school children [28,51,52]. Until now, relatively little attention has been paid to the factors that guide the eating behavior of young adults [53]. However, entering adulthood is a key phase for health. The unhealthy eating behavior that develops at this time can often lead to negative health consequences in the future [28]. The relationship between food and health is a topic of increasing public importance [45]. The mechanisms driving the behavior of young adults should be the foundation for the development of effective strategies to address unhealthy eating behaviors [28]. 

Among the important motivations for adopting healthy eating habits, respondents mentioned the doctor’s recommendations, the intent to lose weight and to live a healthy lifestyle, as well as following a trend. What hinders young Poles from following healthy eating habits is, e.g., time constraints, lack of financial resources, the inability to prepare meals and a lack of knowledge about the principles of healthy eating. According to Odette et al. [54] and Plow and Finlayson [55], the main barrier to healthy eating is the lack of access to healthy food and the need to transport food home, which overlaps with easy access to high-calorie food. These researchers also point out that unhealthy food is available mainly during social gatherings and at work. Plow et al. point to the same problem in their research [56]. In turn, Odette et al. [54] showed that poor housing conditions make it impossible to prepare healthy meals. As in our research, the research of Odette et al. [54], Plow et al. [56], Hall et al. [57] and oraz Nosek et al. [58] showed the barrier of fatigue in preparing one’s own meals. The lack of time is another barrier that prevents healthy eating [56,58], as well as the high cost of healthy food [59,60,61]. 

On the other hand, the barriers to healthy eating mentioned by students in the USA were time constraints and high prices of food products [62], whereas the students surveyed by Sogari et al. [61] also mentioned the lack of motivation to prepare food, snacking, convenient high-calorie food and easy access to fast food. However, the motivations for their healthy eating were better knowledge of food, food education, meal planning, commitment to food preparation, physical activity and the support of friends. These factors are to a large extent a solution to the barriers that prevent the correct eating habits of young adult Poles.

According to Sogari et al. [61], unhealthy eating habits (including lower consumption of healthy foods such as vegetables and fruits, irregular meals, and increased consumption of unhealthy snacks and “junk food”) increase when young adults leave the family home to live alone or with roommates during post-secondary education. A change of environment can strongly affect the forming of new eating habits [62,63,64]. The same experiences can occur when young adults take up jobs and immerse themselves in them to such an extent that they do not have time for proper nutrition. Gall et al. [65] indicate that this phase of life is characterized by a significant increase in autonomy as well as the development of identity and individuality. 

Reducing potential barriers in university campus diners or office centers is recommended as it can contribute to the adoption of better diets by students and young professionals [66]. As, on the other hand, research by Plow and Finlayson [55] and Plow et al. [56] indicates, physicians play an important role in education on healthy eating. In their opinion, doctors should encourage healthy eating and educate on a proper diet. In addition, according to Tomey [59] and Larson et al. [67], the lack of adequate knowledge on nutrition is a significant barrier to healthy eating habits.

Providing young adults with the necessary skills and building their awareness of healthy eating will help them make better food choices later in life. Laska et al. [68] also believe that promoting healthy eating is important at this stage of life. Gomez et al. [69] and Backholer et al. [70] believe that it is imperative to develop programs and tools to increase the motivation of students to choose healthy foods. The above results correspond to the results obtained in the authors’ own research, because the lack of knowledge about the principles of healthy eating is one of the barriers to following a correct diet for young Poles. Social media can also be helpful in promoting a healthy diet [70,71,72,73]. 

Research by Nelson et al. [28], Deforche et al. [72], Finlayson et al. [73] and Niemeier et al. [74] has shown that changes in eating behavior (including skipping breakfasts, snacks, lower fruit and vegetable consumption) contribute significantly to weight gain in people moving from adolescence to younger adulthood. The authors’ own research also showed that the groups with poor and very poor eating habits had the highest percentage of respondents with the level of adipose tissue and BMI indicating overweight and obesity.

The next stage of the research should be to assess whether the specific actions of the Polish authorities, university canteens and workplace canteens are effective in changing behavior towards a healthy lifestyle, especially eating habits.

## 5. Limitation

During the research, the respondents could have been not entirely honest. Thus, there may be a mismatch between stated attitudes and beliefs and actual health behaviors in young Poles. 

## 6. Conclusions

The main reasons for healthy eating among young Poles are: doctor’s recommendations, the intention to lose weight and the willingness to live a healthy lifestyle and to follow a trend. On the other hand, the largest barriers to proper nutrition are: lack of time to prepare healthy meals and of financial resources, inability to prepare meals and limited knowledge of the principles of healthy eating. The eating behaviors varied significantly due to the physical activity of the respondents. Active people’s eating habits were the best, and those of sedentary people the worst. Healthy eaters also had normal body composition indexes (level of body fat, BMI, physique rating). Therefore, eating habits are strongly related to body composition, especially body fat. Therefore, it is important to show this relationship through educational campaigns in various institutions (e.g., schools, universities, workplaces). These institutions should have vending machines with only healthy foods. Canteens should offer balanced whole-week menus tailored to different consumer groups. 

## Figures and Tables

**Table 1:** General characteristics of the study population.

Specification		Proportion in %
Sex	M	43.60
	F	56.40
Place of residence	villages	35.09
	towns with up to 20,000 residents	29.82
	cities with over 20,000 residents	35.09
Region of Poland	north-east	25.06
	north-west	25.06
	south-east	25.06
	south-west	24.82
Professional status	working	25.06
	working and studying	25.31
	only studying	24.57
	secondary school	25.06

**Table 2:** Modifed Starzyńska’s test used to study nutritional behavior.

Specification	Score
The number of meals per day planned in the menu	
4–5	5
3	3
less than 3	0
Frequency of milk or cheese consumption	
daily, in 2 meals	5
daily, at least in one meal and in 50% of days in 2 meals	2
less often	0
Frequency of vegetables or fruits	
daily, at least in 3 meals	5
daily, at least in 2 meals	2
less often	0
Frequency of raw vegetables or fruits consumption	
daily	5
in 75% of days	2
less often	0
Frequency of wholemeal bread, groats and dry leguminous vegetables consumption	
daily, at least in 1 meal	5
in 75% of days, at least 1 of the products mentioned	2
less often	0
Total	25

Source: own study based on [36,37].

**Table 3:** Evaluation of the modified Starzyńska’s test.

Score	Nutrition Assessment
23–25	good
16–22	sufficient
8–15	bad
<7	very bad

Source: own study based on [36,37].

**Table 4:** Logistic regression model (logit)—motivations of young Poles for employing healthy eating habits.

Motivations	*N* = 399; Number 1: 342; Number 0: 57; Total Loss: 144.42; χ^2^ (6) = 98.440; *p* < 0.001						
	Rating	Standard Error	*t* (392)	*p*	χ^2^ Walda	*p* (Dla Walda)	UDR
Constant B0	−4.159	0.877	−4.741	<0.001 *	22.473	<0.001 *	0.016
*x*_1_—doctor’s recommendation *	0.714	0.157	4.549	<0.001 *	20.692	<0.001 *	2.042
*x*_2_—intent to lose weight *	0.647	0.167	3.864	<0.001 *	14.928	<0.001 *	1.909
*x*_3_—intent to live a healthy lifestyle *	0.339	0.150	2.258	0.025 *	5.097	0.024 *	1.404
*x*_4_—following a trend *	0.452	0.172	2.622	0.009 *	6.874	0.008 *	1.571
*x*_5_—persuasion by others	0.096	0.118	0.817	0.414	0.668	0.414	1.101
*x*_6_—lifestyle	−0.149	0. 877	0.759	0.448	0.576	0.448	0.862

* Statistically significant differences (*p* < 0.05).

**Table 5:** Classification of motivations behind healthy eating habits among young Poles.

Observation	Odds Ratio: 45.68%, Correct: 89.72%		
	Predicted No	Predicted Yes	% Correct
no	20	37	35.09
yes	4	338	98.83

Source: own study based on the research.

**Table 6:** Logistic regression model (logit)—barriers for young Poles to employing of healthy eating habits.

Barriers	*N* = 399; Number 1: 342; Number 0: 57; Total Loss: 152.81; χ^2^ (5) = 21.646; *p* = 0.001						
	Rating	Standard Error	*t* (494)	*p*	Walda	*p* (Dla Walda)	UDR
Constant B0	−0.826	0.496	−1.665	0.096	2.773	0.096	0.438
*x*_1_—no time *	−0.369	0.171	−2.159	0.031 *	4.660	0.031 *	0.692
*x*_2_—lack of financial resources *	−0.544	0.151	−3.612	<0.001 *	13.043	<0.001 *	1.723
*x*_3_—inability to prepare meals *	−0.156	0.112	−1.391	0.017 *	1.934	0.016 *	0.856
*x*_4_—lack of knowledge about the principles of healthy eating *	−0.386	0.147	−2.629	0.009 *	6.912	0.009 *	0.680
*x*_5_—no housing conditions	0.073	0.131	0.556	0.578	0.310	0.578	1.075

* Statistically significant differences (*p* < 0.05).

**Table 7:** Classification of cases—barriers to the application of healthy eating habits among young Poles.

Observation	Odds Ratio: 6.12%, Correct: 90.05%		
	Predicted No	Predicted Yes	% Correct
no	4	53	7.02
yes	0	342	100.00

Source: own study based on the research.

**Table 8:** Motivations for the use of healthy eating habits depending on the physical activity of young Poles—discriminant analysis.

Motivations	Model of Discriminant Analysis: Wilks’s λ: 0.571; F(18.110) = 13.416; *p* < 0.001				Classification Functions: Assessment of Physical Activity			
	Wilks’s λ	F	*p*	Tolerance	Inactive *p* = 0.14	Not Very Active *p* = 0.46	Active *p* = 0.35	Very Active *p* = 0.05
doctor’s recommendation	0.635	14.522	<0.001 *	0.890	1.310	2.049	2.292	3.074
intent to lose weight	0.619	10.943	<0.001 *	0.974	1.480	2.196	2.251	3.168
intent to live a healthy lifestyle	0.611	8.961	<0.001 *	0.776	0.668	1.164	1.065	2.081
trend	0.599	6.336	<0.001 *	0. 696	0.121	0.535	0.612	0.271
persuasion by others	0.578	1.461	0.225	0.927	1.043	0.993	1.172	1.208
lifestyle	0.623	11.634	<0.001 *	0.789	1.819	1.281	1.803	2.799
constant					−10.036	−13.616	−17.136	−31.123

* Level of significant difference at *p* < 0.050. Source: own study based on research.

**Table 9:** Barriers for young Poles to employing healthy eating habits depending on the physical activity—discriminant analysis.

Motivations	Model of Discriminant Analysis: Wilks’s λ: 0.824; F(15.108) = 5.213; *p* < 0.001				Classification Functions: Assessment of Physical Activity			
	Wilks’s λ	F	*p*	Tolerance	Inactive *p* = 0.14	Not Very Active *p* = 0.46	Active *p* = 0.35	Very Active *p* = 0.05
no time	0.889	10.165	<0.001 *	0.831	1.146	1.724	1.401	0.388
lack of financial resources	0.866	6.492	<0.001 *	0.792	1.581	0.953	0.954	0.283
inability to prepare meals	0.847	3.620	0.013 *	0.956	1.453	1.627	1.685	1.104
lack of knowledge about the principles of healthy eating	0.880	8.802	<0.001 *	0. 798	0.162	0.522	0.447	1.468
no housing conditions	0.827	0.346	0.792	0.894	1.517	1.487	1.398	1.467
constant					−8.663	−8.610	−7.971	−9.231

* Level of significant difference at *p* < 0.050. Source: own study based on research.

## Data Availability

Not applicable.
